# A Data Fusion Method in Wireless Sensor Networks

**DOI:** 10.3390/s150202964

**Published:** 2015-01-28

**Authors:** Davood Izadi, Jemal H. Abawajy, Sara Ghanavati, Tutut Herawan

**Affiliations:** 1 School of Information Technology, Deakin University, 3220 Waurn Ponds, Geelong, VIC 3216, Australia; E-Mails: dizadi@deakin.edu.au (D.I.); jemal.abawajy@deakin.edu.au (J.H.A.); sghanava@deakin.edu.au (S.G.); 2 Department of Information System, University of Malaya, 50603 Pantai Valley, Kuala Lumpur, 50603, Malaysia

**Keywords:** wireless sensor network, data fusion, fuzzy logic controller, data redundancy

## Abstract

The success of a Wireless Sensor Network (WSN) deployment strongly depends on the quality of service (QoS) it provides regarding issues such as data accuracy, data aggregation delays and network lifetime maximisation. This is especially challenging in data fusion mechanisms, where a small fraction of low quality data in the fusion input may negatively impact the overall fusion result. In this paper, we present a fuzzy-based data fusion approach for WSN with the aim of increasing the QoS whilst reducing the energy consumption of the sensor network. The proposed approach is able to distinguish and aggregate only true values of the collected data as such, thus reducing the burden of processing the entire data at the base station (BS). It is also able to eliminate redundant data and consequently reduce energy consumption thus increasing the network lifetime. We studied the effectiveness of the proposed data fusion approach experimentally and compared it with two baseline approaches in terms of data collection, number of transferred data packets and energy consumption. The results of the experiments show that the proposed approach achieves better results than the baseline approaches.

## 1. Introduction

Wireless sensor networks (WSNs) have been used in various domains such as military applications (e.g., military surveillance) and civil applications (e.g., industrial control and wildlife monitoring). Generally, a large number of sensor nodes, capable of collecting data, processing and communicating among themselves as well as with a base station (BS) are deployed in the sensing field to collect data according to a specific application. The sensor measurements are referred to as a sensor reading or sensor value. The corresponding correct value of an event in the environment is referred to as the true value. If a sensor reading and the true value disagree, the sensor reading is said to be incorrect.

A fundamental issue in WSNs is how various applications such as event detection, target tracking, and decision making can use the sensor measurements with increased confidence and as minimum energy consumption as possible in the presence of imprecise sensor readings. The incorrect sensor readings can be attributed to a number of factors. For example, strong variations of pressure, temperature, radiation, and electromagnetic noise in the monitored area might interfere with the sensor node readings and subsequently could lead to imprecise sensor readings. Furthermore, a sensor node itself might in some cases collect incorrect data due to failure, spatial and temporal coverage problems. Moreover, the neighboring sensors within the sensing field often generate duplicate and highly correlated data, which might also decrease the QoS. In order to overcome these problems, a data fusion mechanism can be used to remove the incorrect and duplicated data from the sensor measurements.

Data fusion mechanisms process the data from multiple sensors and thereby create meaningful new information that cannot be obtained from any single sensor. The main purpose of data fusion mechanisms in WSNs is to provide a greater QoS for the purpose of arriving at reliable and accurate decisions about the events of interest. The QoS here can mean reliable delivery of accurate, complete, and dependable information. In fact, fusing data ensures that not only the data quality of the WSN is enhanced, but also energy consumption can be lowered as it removes redundant information as well.

There are many data fusion mechanisms with the purpose of reducing the energy consumption for WSNs [1–3]. These mechanisms use different techniques such as probability theory [4], fuzzy set theory [5], a combination of fuzzy sets and neural network [6] and Dempster-Shafer evidence theory (DSET) [7]. Most of these approaches are able to eliminate duplicate data in the fusion process. However, these approaches do not consider specific limitations of the sensor devices. For example, they assume that the sensor nodes are always functioning properly and generating accurate data. These are unrealistic assumptions as the environment may change. For instance, the temperature can influence the accuracy of the sensor node functionalities. Moreover, the existing approaches transfer both necessary and unnecessary sensed data to the processing center, which results in excessive energy consumption.

In this paper, a fuzzy-based data fusion approach for WSNs that increases the QoS whilst maximizing the network lifetime by minimizing the energy consumption is presented. By virtue of distinguishing and aggregating only the true values of the sensed data, the proposed approach is able to reduce the transmission as well as the processing of the entire sensed data. It is also able to eliminate redundant data and consequently reduce energy consumption thus increasing the network lifetime. In summary, we make the following contributions:

(a) We propose a data fusion approach to improve the performance of a WSN with respect to the level of QoS generated about the events of interest.
(b) We minimize energy consumption by transferring only the calculated result of the events instead of the entire fused data.
(c) We show that the proposed approach is robust in terms of the events of interest with respect to the sensor node failures as it combines all received data from the sensor nodes.
(d) We studied the effectiveness of the proposed data fusion approach experimentally and compared it with two baseline approaches in terms of data collection, number of transferred data packets and energy consumption. The results of the experiment show that the proposed approach achieves better results than the baseline approaches.

The rest of this paper is organized as follows: Section 2 presents an in-depth analysis of the existing approaches. Section 3 describes the system model and problem overview. In Section 4 the proposed data fusion approach is explained in details. In Section 5 the performance of the proposed approach is analyzed. Finally, the conclusions are presented in Section 6.

## 2. Related Works

In this section, a review of some common protocols that have been proposed to aggregate data is presented. An overview of type-2 fuzzy logic is also discussed. Wireless sensor network applications such as surveillance and reconnaissance generate a large amount of redundant data. There have been many approaches to filter redundant sensing data using data aggregation techniques. These approaches can be classified as cluster-based, tree-based, grid-based and structure-free approaches. A hybrid clustering-based data aggregation mechanism that concurrently combines static and dynamic clustering methods is discussed in [8]. The algorithm chooses a suitable clustering technique based on the status of the network. However, the authors assume that the entire collected data from each sensor node is the true value. That assumption obviously reduces the ratio of packet transmission in the WSN. Another cluster-based data aggregation scheme is discussed in [9]. As the approach is a geographic location-based multicast protocol, data is aggregated based on their geographic locations in their clusters. However, the energy consumption efficiency is not fully addressed as a GPS system is used in each sensor node. Moreover, the sensor nodes simply transfer the data to the base station as the true value.

Tree-based methods arrange the entire sensor nodes into a tree [10]. Xin and Fei-Qi [11] discuss a tree-based protocol where the parent node receives data packets from leaf nodes to aggregate them with the data that is coming from the surrounding node. Then, the parent nodes pass on the new series of data packets to their parent nodes until it reaches the BS. GBDAS [12] is a grid-based data aggregation scheme that partitions the sensor field into a 2-D logical grid of cells. In each cell, the node with the most residual energy (cell head) takes the responsibility for aggregating its own data collected by the other sensor nodes of the cell. The cell heads form a chain and aggregated data moves from head to head along the chain until it reaches the BS. Since all sensor nodes need to aggregate the collected data, the delay especially in the end nodes cannot be ignored. Furthermore, it cannot be a desirable solution for large networks or even for the very far distance sinks.

The dynamic errors or uncertainties related to insufficient or noisy data in many real world applications, can negatively affect the performance of the implemented signal processing systems. There are many techniques that includes statistical and Covariance Intersection (CI)-based methods are used in enhancing the QoS in WSNs. However, they are cannot cope with the uncertainty of the data produced by WSNs. Moreover, the inflexibility of the methods prevents processing the data realistically [13]. In fact, using these methods requires a very complex and substantial computational effort to have optimal performance [14]. A Kalman Filter-based approach to correct the values measured by the sensors is discussed in [15]. Some of these methods are explicitly model-based, whereas others require tuning and training. In the general case, where *a priori* information is often not available, these approaches are typically deficient and can often lead to undesirable results.

Fuzzy logic-based approaches that utilise a fuzzy logic controller embedded in the sensor nodes have been used for cluster-head election [16], for reducing the deadline miss ratio associated with the real-time data transmission from the source node to the actuator maintained at a pre-determined desired level [17] and online routing algorithms to address the maximum lifetime routing problem in wireless sensor networks [18]. The distinguishing aspect of our work is the novel use of fuzzy membership functions and rules in the design of cost functions for the routing objectives considered in this work. In [15], a variable weight-based fuzzy data fusion algorithm is proposed. The main purpose is to enhance the accuracy of the data fusion process in WSNs. In this approach, each CH is assigned a different and unfixed fusion weight. The weights are changed using a fuzzy logic system, based on some factors such as delay, data amount and trustworthiness of the CHs. Lower fusion weighted CHs have lower influence. However, the authors did not take the message overhead and energy consumption into account. Shell *et al.* [13] presented a fuzzy logic system that is able to isolate instances of failure within the defined data set with fewer perceived instances of false positives and a higher degree of accuracy than classical methods alone. However, the authors did not consider the energy consumption properly.

In this paper, we use an interval type-II fuzzy logic system (T2FLS) as opposed to a type-1 fuzzy logic system (T1FLSs). The fuzzy membership functions in T2FLSs use membership degrees that are themselves fuzzy sets, whereas T1FLSs use fixed fuzzy memberships that cannot directly address variable conditions. Therefore, uncertain measured parameters would be neglected by a T1FLS and the performance will obviously be negatively influenced. This makes type-2 fuzzy sets very useful when there is difficulty in deciding the appropriate membership function with ambiguity. A recent study that compared the effects of the measurement noise in type-1 and type-2 FLSs concluded that the use of T2FLSs in real world applications that exhibit measurement noise and modelling uncertainties can be a better option than T1FLSs [17,19]. Furthermore, T2FLS technology has been regarded as a way to increase the fuzziness of a relation which implies an increased ability to handle inexact information in a logically correct manner [18].

Although there are works such as Z-slices [20], *α*-planes [21,22] or *α*-cuts [23] that use T2FLS or generalized T2FLS, interval T2FLS is preferred for use due to the computational complexity of the former [24]. The computations associated with IT2 fuzzy sets are very manageable, which makes interval T2FLS quite practical [25]. The basic concept of interval T2FLS is to consider a footprint of uncertainty, which can be described by two parameters bounding T1 fuzzy membership functions [18]. The interval T2FLS Ã can be calculated as follows:

(1) $$\tilde{\text{A}}=\iint \begin{array}{cc} \frac{1}{\left(\text{x},\text{u}\right)} & {\text{J}}_{\text{x}}\text{C}\;[0,1] \end{array}$$

where x and u are the primary and the secondary variable and J_x_ is the primary membership function of x. In case of IT2 fuzzy sets, all secondary grade of fuzzy set Ã are equal to 1. The domain of the primary membership J_x_ defines a footprint of uncertainty of fuzzy set Ã, which can be described by its upper and lower membership functions. Hence, a footprint of uncertainty (Ã) = U_x∈X_( μ_Ã_(x), (μ̄_Ã_(x)).

## 3. System Model and Problem Overview

In this section, we present a brief description of our WSN system model and also present the problem overview. We consider a WSN with a large number of sensors distributed randomly in a certain deployment area. The sensors are organized into clusters based on their spatial proximity and each cluster has a CH [26]. There are three types of sensor nodes that collect temperature, humidity and smoke density data and then send it to its CH. The CHs are responsible for fusing and transferring the data to the BS. Then, each node, based on their current situation, uses FLC to assign a weight for their data. Next, the data are sent to the CH, which is connected to a BS wirelessly.

Uncertainty inherent in sensor measurements is one of the most fundamental issues to be addressed in a WSN environment. As sensor nodes are typically deployed densely, there exists significant redundancy in the data collected from sensor nodes. The duplicate data may lead to serious packet collisions, bandwidth waste and energy consumption. Similarly, sensor nodes can report erroneous readings for a number of reasons such as a possible manufacturing defects or environmental conditions. As a result, they can negatively affect making accurate decisions as well as the efficiency of energy consumption in the WSN. To solve the problem, it is necessary to address the unnecessary data in addition to redundant data transferred from sensor nodes.

**Figure 1.**
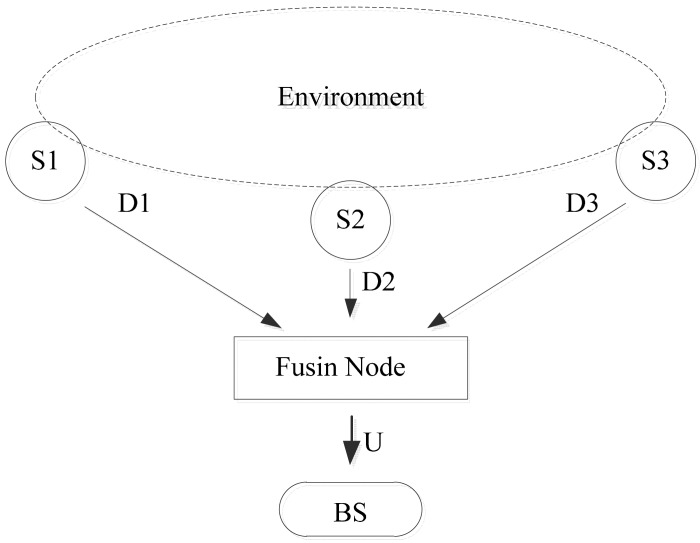
Fusion mechanism.

These uncertainties necessitate the development of sensor data fusion strategies that can combine information in a coherent and synergistic manner to yield a robust, accurate, and consistent description of the quantities of interest in the environment. A general block diagram of a data fusion mechanism is given in Figure 1. The sensor nodes *s*_1_, *s*_2_ and *s*_3_ collect data *D*_1_, *D*_2_ and *D*_3_ from the environment. In many situations that depend on the current conditions of the sensor nodes, *D*_1_, *D*_2_ and *D*_3_ might not be exactly the true value. There are many useless data packets generated and transferred from each sensor node in each round of data collection. In many situations, a sensor node is not able to recognize the useless data while it generates data packets for further processing.

Rather than each sensor node sending the data to BS, they send the data to a fusion node. The fusion node creates a single internal representation of the environment from its inputs. The single representation is then forwarded to the BS. This means that the BS in general does not have access to the individual sensor measurements.

The main cause that negatively influences performance of a data aggregation mechanism is misbehaving sensor nodes. Different environmental factors such as a sudden change of temperature or humidity can influence a sensor node's behavior. These environmental factors decrease or increase the output signal of the sensors, which creates an ultralow frequency noise in the transferred signals. In addition, non-operating environmental limits such as a high or low temperature of air surrounding the sensor nodes usually influence the sensor's performance. The operating temperature range is the length of ambient temperatures given by their upper and lower extremes, within which the sensor nodes maintain their expected accuracy. In different WSN applications, it is impossible to confirm that the collected data are true values of the events without taking samples or analyzing the data history. Sensors usually carry noise in their measurements, thus we cannot be 100% sure that the measured value is correct. It does not matter how an event (e.g., temperature) is measured by a sensor or how close the measurement is to the true value, we can never be sure that it is accurate. Equation (2) captures the error induced by a sensor node [27]:

(2) $$\phi =t^{\prime }-t$$

where *t* is any individual measurement and *t′* is the true value.

Let *R* be the percentage of the correct data received by BS. Equation (3) shows the percentage of the accurate data packets received by a BS:

(3) $$R=\frac{\upsilon_{p}}{L}\times 100$$

where *L* is the total number of events defined by the application and *ν_p_* is the number of messages that contain only the true value of the received data.

Let Z_c_ be the network lifetime, *RD* be data redundancy that is satisfied by a user-defined value (*RD_max_*) similar to the percentage of collected true value of data (*RD_min_*). The problem addressed in this paper is formulated as follows:

(4) $$\text{Maximise}\;{\text{Z}}_{\text{c}}$$

(5) $$\text{s}.\text{t}.\;R\geq R_{\text{min}}\;\text{and}\;RD\geq RD_{\text{max}}$$

The objective is to maximize the network lifetime Equation (4) ensuring that the percentage of the true value of data and data redundancy are satisfied by a user-defined value (Equation (5)). Since it is impossible to confirm that the collected data are true values of the events without taking samples or analyzing data history [26], we suggest assigning a weight for each collected data. The weights are determined based on current storage conditions of the source nodes. If the senor nodes are not in the expected condition as revealed in their data sheets, the determined weights are changed. The better condition a sensor node is the higher weight is assigned to its collected data. In fact, the weights show the percentage of correctness of the data. Based on the calculated weights, the correct value of data can be distinguished and separated from others. As a result, a higher QoS can be received by the BS.

## 4. Proposed Data Fusion Approach

Algorithm 1 explains the proposed data fusion process. This algorithm is collecting data from the three different sensors until it detects the events. The inputs of the algorithm are node temperature, humidity ratio and signal to noise ratio. The output of is the fused collected sensory data.

**Algorithm 1** Data Fusion Algorithm
1.**INPUT**: (T: Node temperature, H: Humidity ratio, N: Signal to noise ratio)2.**OUTPUT**: Fused Data3.**BEGIN**4.**WHILE (Event NOT Detected)**5.**FOR** all the cluster members in one kind6. **CF_n_** ← FLC (T, H, N)7. **IF CF_n_** ≥ ***δ***8.  Data and **CFn** will be sent CH9. **ELSE**10.  Collected data will not be considered11. **END IF**12.**END FOR**13.**FD_n_** ← Received data by CHs from one kind of node will be fused14.Consequent ← FLC (**FD_1_**, **FD_2_, …, FD***_n_*)15.**IF** the consequent was not changed16. Disregards the received data17.**ELSE IF**18. Send the Consequent to BS19. **IF** the event detected by BS20. Report the event21. **END IF**22.**END IF**23.**END WHILE****END Algorithm**


**Figure 2.**
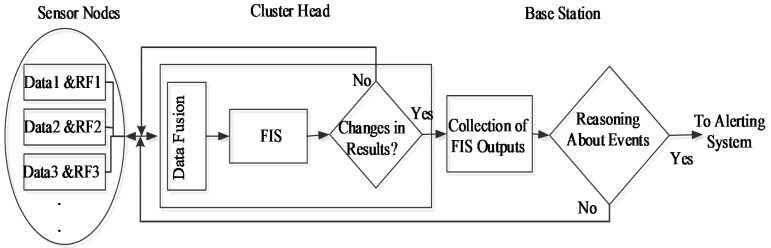
Proposed flow chart.

Figure 2 shows the proposed data fusion and transferring process. As in previous works [15,27–29], the sensors will be embedded with a fuzzy logic controller (FLC). The purpose of FLC is to find a confidence level of the collected data considering the current condition of the sensor nodes. Each sensor node collects data and calculate a confidence factor (CF) using FLC for each collected data packet. To calculate CF, the FLC is developed based on the Sugeno method [30].

The FLC considers the non-operating temperature (T) and humidity (H) range to create the membership functions. A random membership function is also used for noise to signal ratio (N) of the sensors. FLC produces a confidence factor (*CF*_1_, *CF*_2_, …, *CF_n_*) for each sensor data (*D*_1_, *D*_2_,…,*D_n_*). There are three inputs for the FLC: Temperature, Humidity Rate and Signal to Noise Ratio. For each input synthetic data is used. For each data we use a Gaussian distribution with its mean and covariance matrix representing the expected value and its uncertainty (10% of the value). Then, the values are normalized to fit in the [0,1] as the inputs of the fuzzy system. Then, we extracted linguistic variables from the normalized data. The linguistic variables used to represent them are divided into three levels: Low, Medium and High. The consequent or the output of the FLC is divided into five levels: Very Low (VLow), Low, Medium, High and Very High (VHigh). 20% of the data is used to determine the membership functions and also the rules.

**Figure 3.**
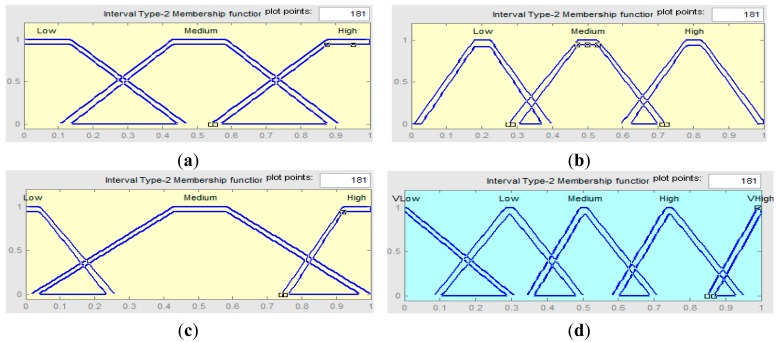
Membership functions of FLC. (**a**) Temperature; (**b**) Humidity Rate; (**c**) Signal to Noise Ratio; (**d**) Confidence Factor.

Based on the fuzzy variables shown in Figure 3, fuzzy rules are defined as shown in Table 1. Since each input variable has three fuzzy states, e.g., Low, Medium and High, thus, the total number of possible fuzzy inference rules for the developed system, is 3 × 3 × 3 = 27.

The FLC determines whether the temperature and humidity rates of the sensor nodes and also the signal to noise ratio are in the acceptable range. To accomplish that, the FLC compares its input measurements with the desired range for each sensor. The desired range for each sensor can be found on its specified datasheet. The output of FLC for each sensor can be 100% only if the environmental factors are in the desirable range.

In case of being out of the range, FLC produces a confidence factor (0% ≤ *CF_n_* < 100%) for the collected data. Each node compares the calculated *CF*s against a cut-off value. In the proposed system, the value is provided by users to decide if the fuzzy output should be considered. If the created factor of each data is less than the considered value, the data will be disregarded. Otherwise, it will be sent to CHs in a data message. This prevents the corruption of the correct value of data by the other data that does not present the true values. For fusion purpose, the message also consists of a Node-ID of each source node.

CHs aggregate the data received from the cluster members. The data fusion process is started by CHs at the end of each round of data collection. Equation (6) is used to aggregate the entire data received from cluster members in the same kind with different locations:

(6) $$FD=\frac{\left(CF_{1}\times D_{1}\right)+\left(CF_{2}\times D_{2}\right)+\left(CF_{3}\times D_{3}\right)+\cdots +\left(CF_{n}\times D_{n}\right)}{CF_{1}+CF_{2}+CF_{3}+\cdots +CF_{n}}$$

where *D_n_* is the received data from the same kind of cluster members (e.g., light detector), *CF_n_* is the calculated confidence factor of the collected data by each sensor node and *FD* is a combination of the data received from them. In fact, *FD* is a combination of the data with a higher certainty as it combines the received data based on their confidence factors. As a result of considering different sources of one kind of sensor node in different locations, *FD* provides a better view of the environment. The *FD* is also robust as data from multiple sensors with their own confidence factors mitigate the problem of sensor failure. That is because in case of any disorder of a sensor in a cluster, the environment still can be monitored as the entire received data is combined and then examined by the system. The *FD* is calculated for any kind of deployed sensor nodes individually. Therefore, that would be a set of *FD*s. Next, *FD*s are stored in a matrix with one row and m columns (*V_FD_*). Equation (7) presents *V_FD_* matrix:

(7) $$V_{FD}=\left\{FD_{1},FD_{2},\ldots ,FD_{m}\right\}$$

As an example, we consider three temperatures, four light and three smoke density sensors. They are deployed on a cluster-based method. Over a period of time, the temperature sensors sense the environment to be at 20 °C, 15 °C and 10 °C with the confidence factors of 0.75, 0.65 and 0.41 respectively. As a result, the fused temperature data (*FD_T_*) would be 15.93. The fused light detector (*FD_L_*) and fused smoke density data (*FD_s_*) are 49.8 and 33.2, respectively. Based on this *V_FD_* matrix of the monitoring system is *V_FD_* = {15.93, 49.8, 32.2}

Then, the vector *V_FD_* will be fed and processed by the FLC with different inputs in the CHs. The output of the system is the consequent (fuzzy response) of events occurring in the monitored areas. FLC analyses the *V_FD_* vector based on a provided fuzzy rules after data fusion process was accomplished. If the consequent of the events in the clustered nodes was not changed, the CHs do not forward the data packet to the BS. Otherwise, the CHs send the output of the system to the BS. However, the change in the system output does not guarantee a correct detection and it is only considered as a possible event in the monitored area. Therefore, to make sure that the detection is accurate enough, the BS needs to regularly monitor and process the received outputs that are generated over the time. In fact, all the received probabilities are constantly processed by the BS. If a constant change is noticed, the event will be reported to the alerting subsystem by BS. Otherwise, the algorithm continues collecting data.

## 5. Performance Analysis

In this section, we present the performance analysis of the proposed approach and compare it with two other approaches. In the experiment, we focus on the expected amount of colleting correct data with respect to QoS.

### 5.1. Experiment Setup

We model the MTS420/400 sensor board and an IRIS with ATmega1281 processor and a mib520 programming base as BS. The sensor board consists of humidity, temperature and light sensor as well as a communication component. Table 2 presents the information about MTS420/400. In order to simulate noise effects in real sensors, a random high-frequency noise signal is added to the sensor signal. MATLAB was used to analyse the performance of the network.

We calculate the energy consumption in each round of data collection using the method proposed in [31]. We use Equation (8) to evaluate the traffic flow of each protocol:

(8) $$\text{Trafffic overflow}=\frac{\text{transferred data packets}}{\text{generated data packets}}$$

The proposed approach is compared against forest fire detection (FIM) [32] and a variable weight-based fuzzy data fusion algorithm for WSNs (VWFFA) [15] algorithms. The main reason for choosing the FIM is because it is a distributed algorithm that uses MTS420/400C sensor boards for the proposed algorithm. The algorithm is based on a state machine with five states. The transition from one state to another is generated when a relevant change is detected, indicating the probable existence of an event. If the ratio is less than a defined value, the machine moves to another state. The authors proved that algorithm based on a defined cut-off value method can be implemented more easily and with better performance than an algorithm based on Dempster-Shafer theory [32]. VWFFA is also chosen because it is a distributed fuzzy-based data fusion algorithm that enhances the QoS in WSNs. It is a weight-based fuzzy data fusion algorithm for WSNs that improves the accuracy and reliability of the global data fusion. In this algorithm, the weight of each CH in the global fusion is not fixed. The time delay, amount of data and trustworthiness of each CH affect the final fusion weight. That means a CH with too small a data value or too low trustworthiness cannot be given a big fusion weight. As a result, CHs with deficiencies have a small influence in global fusion. For validation purposes, we used the root means square error (RMSE). Table 3 compares RMES of the proposed approach via FIM and VWFFA. To calculate RMSE, the most optimum result (100%) is considered as the predicted result in each call.

### 5.2. Result and Discussion

**Figure 4.**
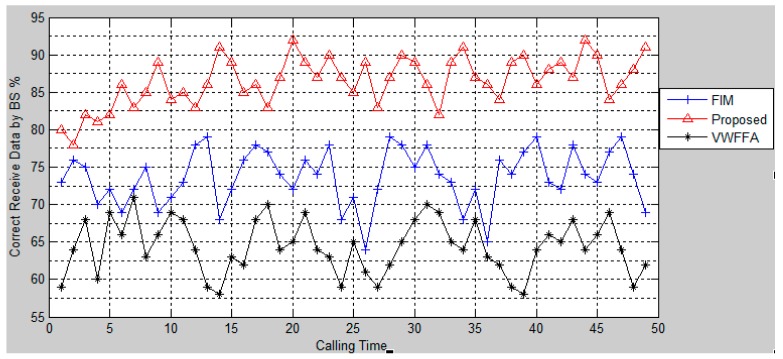
Data collection.

Figure 4 presents a percentage of correct data collected by BS by the proposed approach as well as FIM and VWFFA. As can be seen from the figure, the proposed approach provides a higher percentage of correct data compared with the other approaches.

That is because the proposed approach eliminates the incorrect data to prevent corrupting the true value data in the fusion process. The result also shows that FIM has better performance than VWFFA. This is due to the consideration of uncertainty in the detection process. VWFFA provides the worse performance. In VWFFA the quality of collected data is considered based on their assigned weights. However, the weights are considered on all the data that includes true values and incorrect values of the data collected. Since the entire received data are aggregated by CHs, the true values might be influenced by the incorrect data in the fusion process

**Figure 5.**
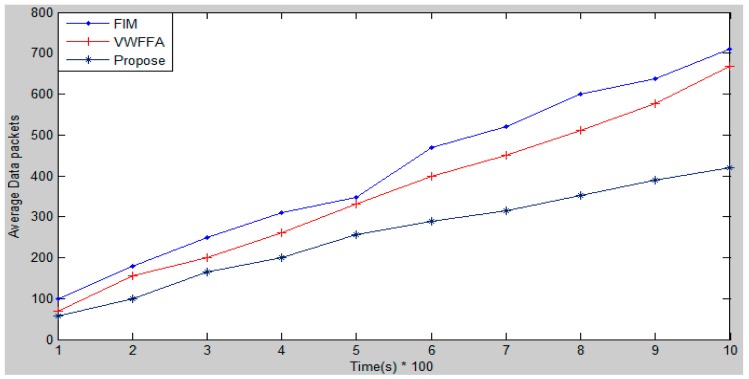
Transferred data packets.

Figure 5 shows the average number of the data packets that are transferred. The proposed approach has a lower average number of the data packets that are transferred than that the FIM and VWFFA. The proposed approach has better performance than the other approaches because the entire collected data do not need to be transferred as only the true valued data are transferred. Moreover, CHs are not required to transfer the fused data as they are able to find the consequent result of the occurring events. The CHs send the calculated consequent only if it shows an abnormality based on the history of the data, so the data that is sent to the BS is going to be only the possibility of the detected fire. In contrast to the proposed approach, FIM has the highest number of data packets that need to be transferred in the network. The reason behind that is the entire incorrect and correct collected data and also redundant data need to be transferred. VWFFA has a lower transferred data number in the network than FIM. The reason behind that is the developed network is cluster-based and the data are fused based on the assigned weights on clusters. In VWFFA, the assigned weights reduces the influence of the incorrect data on the true values in the fusion process.

**Figure 6.**
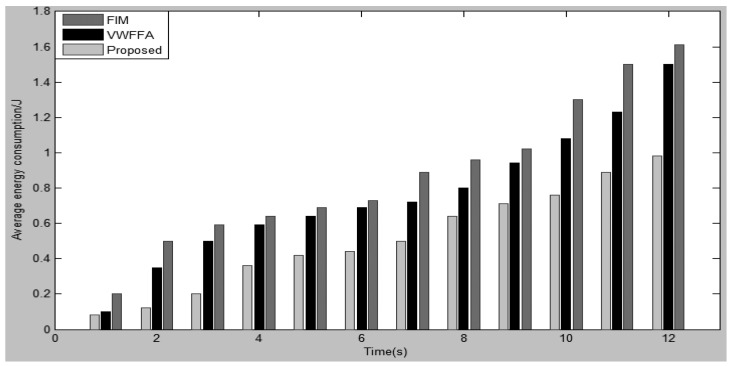
Energy consumption.

Energy consumption is also a critical feature of a WSN. The study by Hill *et al.* [32] proves that each bit transmitted in a WSN consumes about as much energy as executing 800–1000 instructions. That means any reduction of the data transmitted in the WSN can significantly decrease the energy consumption. Figure 6 illustrates the energy consumption in the networks. From the figure, it can be seen that the proposed scheme minimizes the energy consumption in WSN. That is due to the lower transfer data packets in the network (Figure 5). Moreover, data packets similar to VWFFA are sent to their CHs consequently, lower energy is required as compared to FIM.

## 6. Conclusions

Handling sensing data errors and uncertainties in WSN while maximizing network lifetime are important issues in the design of applications and protocols for wireless sensor networks. In this paper, we have presented a fuzzy-based method for data fusion. Through experiments, we show that the proposed approach is able to distinguish and aggregate only true values of the collected data and is able to eliminate redundant data and consequently reduce energy consumption thus increasing the network lifetime. Performance analysis of the proposed approach shows that the algorithm is effective in the quality of the data fusion. The proposed algorithm is compared against two baseline approaches experimentally in terms of data collection, number of transferred data packets and energy consumption. The results of the experiment show that the proposed approach achieves better results than the baseline approaches. In the future we will study the channels and related issues that might occur in transferring data from the CHs to the BS. We will also consider over the air programming to dynamically update the deployed nodes in the area of interest.

## Figures and Tables

**Table 1.** Inference rules.

**Rule NO**	**Input Variable**			**Output**

	**Temperature**	**Humidity Rate**	**Signal to Noise**	**Cofidence Factor**
1	High	High	High	VHigh
2	High	High	Medium	VHigh
3	High	High	Low	High
4	High	Medium	High	High
5	High	Medium	Medium	High
6	High	Medium	Low	Medium
7	High	Low	High	Medium
8	High	Low	Medium	Medium
9	High	Kow	Low	Low
10	Med	High	High	Medium
11	Med	High	Medium	Low
12	Med	High	Low	Low
13	Med	Medium	High	Medium
14	Med	Medium	Medium	Medium
15	Med	Medium	Low	Low
16	Med	Low	High	Medium
17	Med	Low	Medium	Low
18	Med	Low	Low	VLow
19	Low	High	High	High
20	Low	High	Medium	Medium
21	Low	High	Low	Low
22	Low	Medium	High	Medium
23	Low	Medium	Medium	Low
24	Low	Medium	Low	VLow
25	Low	Low	High	Low
26	Low	Low	Medium	VLow
27	Low	Low	Low	VLow

**Table 2.** Parameters of MTS420/400.

**Parameters**	**MTS420/400**
Temperature Range	−40 to +123.8 °C
Humidity Range	0 to 100% RH
Signal to Noise Ratio	0 to 1

**Table 3.** RMSE.

**Approaches**	**RMSE**
Proposed approach	3.67
FIM	5.13
VWFFA	5.9
